# Advanced Materials and Processing Technologies

**DOI:** 10.3390/ma19071276

**Published:** 2026-03-24

**Authors:** Lidija Ćurković, Sonja Jozić, Irena Žmak

**Affiliations:** 1 Department of Materials, Faculty of Mechanical Engineering and Naval Architecture, University of Zagreb, Ivana Lučića 5, HR-10000 Zagreb, Croatia; 2 Department of Manufacturing Engineering, Faculty of Electrical Engineering, Mechanical Engineering and Naval Architecture, University of Split, Ruđera Boškovića 32, HR-21000 Split, Croatia; sonja.jozic@fesb.hr

This reprint gathers the fourteen research articles originally published in the Special Issue of *Materials* titled “Advanced Materials and Processing Technologies”. Additionally, it includes an editorial that places the contributions within the broader context of current research, highlighting recent advancements, existing knowledge gaps, and promising directions for future studies across the covered topics. The contributions cover a wide range of topics, from thermochemical surface treatments such as nitriding and micro-arc oxidation to laser- and plasma-assisted modifications of cast iron and magnesium alloys. They also include the mechanics of adhesively bonded carbon fiber-reinforced polymer joints and the interfacial chemistry of ceramic-to-metal connections. The scope further encompasses additive manufacturing of architected polyamide structures, surface wettability control for thermal management, and the physics of strongly correlated electron systems. The reprint addresses challenges in sustainable technology, including recycling and additive manufacturing of NdFeB permanent magnets, hydrometallurgical gold recovery from electronic waste, bio-based agricultural aggregates in construction, and thermo-mechanical degradation of turbine components. Overall, these works highlight the core idea of modern materials research: that processing methods, often more than composition alone, influence functional behavior, and progress depends on deliberately integrating structural understanding across multiple scales.

Nitriding is a thermochemical surface treatment widely used to enhance the mechanical and corrosion properties of steel, and there is growing interest in sustainable, environmentally friendly approaches such as non-toxic, cyanide-free salt baths and low-temperature processes. In one study, a non-toxic KNO_3_ + KCl salt bath at 650 °C for 3 h produced an approximately 8 μm white layer on low-carbon steel and increased surface hardness from 198 HV to 702 HV, demonstrating a cleaner production route [1]. Similarly, salt bath nitriding of AISI 316L at 580 °C for 10 h produced expanded austenite, increasing surface hardness sixfold to approximately 1100 HV and improving wear resistance [2]. In a related approach, nitrogen diffusion profiles and hardness gradients in gas-nitrided 16MnCr5 steel were quantified across 510–590 °C, providing benchmarks for diffusion-zone design [3]. However, low-temperature plasma nitriding of AISI 316L at 430 °C increased surface hardness by forming expanded austenite, whereas longer treatments led to brittle nitride precipitates that reduced corrosion resistance [4]. A review of low-temperature plasma, implantation, and gas nitriding methods concluded that these treatments create nitrogen-rich diffusion zones that harden surfaces while retaining core properties [5]. Likewise, an optimized three-step gas nitriding cycle for 33CrMoV12-9 steel yielded a surface hardness of 810 HV and a nitrogen diffusion depth of approximately 0.7 mm, without the formation of complex nitride networks [6]. Another study showed that KNO_3_-based salt-bath nitriding of 20MnCr5 steel at 600 °C for 3 h increased surface hardness by about 80% and decreased the corrosion rate by roughly 58% in 3.5% NaCl [7]. Finally, it was concluded that low-temperature thermochemical treatments create expanded phases that increase surface hardness to about 1400 HV, enhance wear and corrosion resistance, and prevent chromium depletion [8].

The technological effort to improve the durability of cast iron parts against cavitation and erosive wear has led to a variety of surface modification methods, ranging from chemical coatings to advanced laser treatments. To find direct solutions for engine parts, researchers combined electroless Ni-P plating with high-temperature heat treatment to greatly increase the cavitation resistance of gray cast iron cylinder liners [9]. Supporting these treatment studies, a basic analysis of vibration-induced cavitation shows how piston slaps cause mechanical stresses that require such protective surface treatments in diesel engines [10]. A review emphasizes that surface modification, including techniques such as TIG remelting and nitriding, remains the primary means of improving the resistance of metal parts to ultrasonic cavitation [11]. Focusing specifically on HT200 gray cast iron, one study shows how laser cavitation peening with copper coverage layers can enhance surface integrity and generate beneficial residual stresses [12]. Further research into laser-based mechanical impacts indicates that extensive laser cavitation peening refines grain structures in HT250 gray cast iron and boosts its electrochemical corrosion resistance [13]. Moving on to high-energy beam techniques, experiments with laser surface melting and alloying on vermicular cast iron demonstrate success in altering microstructures to enhance resistance to aggressive erosion [14]. Comparative studies on austempered ductile iron show that controlling laser fluence can transform the ausferritic matrix into hardened martensitic structures with complex carbides [15]. Optimizing induction hardening parameters, such as power and treatment time, can customize the wear properties of nodular cast iron by enhancing surface hardness [16]. Beyond surface treatments, the mechanical properties of very large cylinder liners can be improved by adding vanadium, which enhances the pearlite matrix and sheet spacing [17]. A comparative analysis of induction hardening, plasma nitriding, and TIG remelting found that local surface remelting offers the greatest improvement in cavitation erosion resistance for EN-GJL-200 gray cast iron [18]. Lastly, when evaluating conventional slurry applications, a study on white cast irons shows that both commercial and alternative heat treatments are essential for optimizing microstructure to resist erosive wear in pumping systems [19]. The combination of multi-step surface modification techniques and precise alloying facilitates the future development of cast iron materials that can withstand the increasingly harsh operational conditions of modern industrial machinery.

The development of surface modification techniques, especially micro-arc oxidation and related hybrid processes, is a key area for enhancing the performance of magnesium alloys for various biomedical, marine, and industrial applications. Researchers developed a multifunctional coating combining Prussian blue and Mg-Zn-Fe layered double hydroxide composite coating that improves the corrosion resistance, osteogenic activity, and near-infrared-triggered antibacterial properties of pretreated magnesium alloy substrates [20]. By using a multi-step approach that involves growing zeolite imidazolate framework in situ, which is a specialized type of metal–organic framework, and then fluorosilanization, a durable superamphiphobic coating system was made that increases corrosion protection by four orders of magnitude and makes it 34 times longer for ice to form in marine settings [21]. The integration of CO_2_ gas during the initial stages of micro-arc oxidation, followed by hydrothermal strontium incorporation and poly(lactic-co-glycolic acid) sealing, a biodegradable polymer, as a final physical barrier layer in a hierarchical coating system, creates a hierarchical defense system that slows down the degradation of biodegradable magnesium-based bone implants [22]. Investigation of physical processing parameters shows that the combined use of ultrasonic vibration and increased current-pulse frequency effectively reduces microdefects and surface roughness, enhancing the wear and corrosion resistance of magnesium–lithium alloys [23]. The creation of dual structures, in which layered double hydroxide nanosheets fill the natural volcanic pores of a middle zeolite imidazolate framework layer, results in superior barrier performance against corrosive media leaking onto magnesium alloy AZ31 substrates [24]. Moving toward sustainable manufacturing, a fast, low-voltage manganese-vanadium-phosphorus electroconversion coating offers a green, energy-efficient alternative to traditional anodizing, creating a compact, protective film with superior salt-spray resistance [25]. A systematic review emphasizes that, although micro-arc oxidation coatings are naturally limited by porosity, their protective effectiveness can be strategically enhanced through specific pre-treatments, such as laser melting, and post-treatments, such as hydrothermal sealing [26]. Experimental analysis shows that Ti-reinforcement phases affect micro-arc oxidation growth by inducing intense localized discharges, leading to a dark blue composite ceramic coating containing titanium dioxide [27]. Researchers engineered the electronic properties of surface layers by using zirconia nanoparticles to modify layered double hydroxide/MgO composites, creating a semiconducting barrier with a low defect density that effectively traps chloride ions, thereby improving corrosion resistance [28]. These combined advancements indicate a future in which magnesium protection strategies will increasingly depend on hierarchical, intelligent, and eco-friendly hybrid systems designed to meet the rigorous chemical and mechanical requirements of complex environmental conditions and applications.

Modern structural engineering increasingly relies on carbon fiber-reinforced polymer bonding to enhance the strength and durability of lightweight components across the aeronautical, automotive, and civil sectors. A foundation for this field is provided by a synthetic review that summarizes various joint configurations and predictive modeling techniques, such as cohesive-zone modeling, to inform design decisions [29]. Based on these fundamentals, researchers have developed a universal analytical solution to predict bond behavior in double-strap joints by explicitly accounting for the effect of steel yielding on stress distributions [30]. Further parametric studies show that tough, ductile adhesives often outperform traditional rigid options when strengthening metallic infrastructure [31]. The strategic use of multiple adhesives with the bi-adhesive technique can further enhance damage tolerance and energy absorption in single-step joints [32]. However, these configurations must endure complex mixed-mode loading, in which even small initial peeling angles can reduce the joint’s initial shear load capacity [33]. In addition to external loads, the presence of unavoidable bondline defects, especially multiple small flaws, reduces the overall joint capacity [34]. For specialized materials such as three-dimensional fiber metal laminates, advanced numerical methods are necessary to capture complex failure modes, such as core delamination and strap debonding [35]. Innovations in additive manufacturing also introduce new variables, such as printing angles, which influence interlayer adhesion and the ultimate strength of 3D-printed polycarbonate joints [36]. Environmental durability poses another challenge, as shown by the decline in the mechanical properties of composite joints after long-term seawater immersion in maritime conditions [37]. Optimizing the geometry of the bonding area, including overlap length and adherend thickness, remains essential for smoothing stress distributions and increasing the stiffness of common single-lap joints [38]. Advanced surface engineering, such as selective UV picosecond laser treatment, provides a precise means to remove contaminants and expose carbon fibers, thereby improving interfacial shear strength [39]. To assess these treatments, mechanoluminescence technology provides a unique way to visualize interfacial strain distributions and track crack propagation in real time [40]. Lastly, external reinforcements, such as scarf and stepped repairs, have proven to be highly effective strategies for restoring strength to damaged aircraft composite structures [41]. As bonding technologies continue to advance, future research should incorporate these innovations in surface engineering and real-time structural health monitoring to ensure the long-term reliability of hybrid material systems.

Based on these advances in joint configurations, surface engineering, and environmental resilience, the following section narrows its focus on the specific mechanics and optimization strategies that govern adhesively bonded carbon fiber-reinforced polymer single-lap joints. This is where laminate architecture, surface functionalization, and geometric parameters converge to determine structural performance. The continuous pursuit of lightweight, high-performance hybrid structures has driven extensive research into optimizing the mechanical integrity, durability, and resilience of adhesively bonded carbon-fiber-reinforced polymer single-lap joints. Research into internal laminate design shows that double-double layups can improve structural response, increasing damage onset force by up to 25% compared to traditional laminates through better material homogenization [42]. Similarly, using multiple repeated plasma surface treatments provides an effective chemical functionalization method, where twenty repeats can increase Mode I specific fracture energy by nearly 20 times compared to untreated joints without changing surface roughness [43]. During the manufacturing phase, optimizing cutting parameters is important for ensuring the reliability of mechanical assemblies, as polishing drill bits to reduce friction has been shown to effectively reduce tool wear and thermal degradation during the stack drilling of carbon-fiber-reinforced polymer and AA7075 aluminum alloy [44]. When optimizing joint geometry, numerical simulations indicate that the optimal adhesive thickness typically ranges from 0.1 to 0.3 mm, as thicker bondlines amplify peel stresses and rotation-induced degradation [45]. Under long-term cyclic loading, geometric factors like overlap length and bondline thickness affect fatigue life, with the Smith–Watson–Topper criterion proving to be the most precise model for predicting high-cycle performance [46]. The vulnerability of these structures to accidental damage is emphasized by the fact that low-energy transverse impacts cause delamination, resulting in a substantial reduction in residual tensile strength [47]. To reduce losses caused by impact, implementing double-adhesive joints with ductile adhesives at overlap ends and brittle adhesives in the middle has proven to be effective in redistributing shear stresses and preserving overall structural capacity after an impact [48]. These advancements in material architecture, surface functionalization, and geometric optimization lay a strong foundation for the next generation of durable hybrid structural joints capable of enduring extreme mechanical and environmental stressors.

Achieving reliable bonds between fundamentally dissimilar materials, such as ceramics and metals, presents a completely different set of interfacial challenges that require specialized alloying and advanced processing strategies. The effort to achieve reliable ceramic-to-metal connections has driven the development of new strategies, including active element doping, ultrasonic assistance, and specialized heating techniques, to address the inherent non-wettability of these dissimilar materials. By incorporating trace amounts of titanium (0.2 wt.%) into Sn-Ag-Cu alloys, Liu et al. have demonstrated that ultrasonic vibration can effectively enhance the metallurgical bonding between ZrO_2_ and stainless steel, even at low concentrations of reactive elements [49]. Similarly, ultrasound can be used to activate commercial inactive solders by promoting the intense dissolution of aluminum from a 6063 aluminum alloy substrate, which then reacts in situ to form a stable amorphous Al_2_O_3_ bridge with SiC ceramics [50]. For specialized structures, sonocapillary action, a phenomenon observed in ultrasonic-assisted soldering, enables Sn-9Zn solder to quickly infiltrate the microchannels of porous Si_3_N_4_, ensuring a dense bond without flux or active fillers [51]. Moving away from mechanical agitation, adding rare-earth elements to Sn-Bi matrices promotes strong bonding between bare glass and copper by forming thin interfacial oxide layers under inert-atmosphere conditions [52]. Further investigation into the combined effects demonstrated that the combination of titanium and magnesium in Bi-Ag-based solders enables SiO_2_ glass and stainless steel to be bonded simultaneously, with titanium predominantly reacting at the steel interface and magnesium accumulating at the glass interface [53]. Emerging technologies such as indirect electron-beam heating have also been successfully applied to Bi-Ag-Ti systems to produce high-strength SiC joints by optimizing wettability at elevated temperatures of 950 °C under vacuum [54]. To address the challenge of thermal expansion mismatch, the incorporation of B_4_C particles into Ag-Cu-Ti metallization layers creates a gradient structure that effectively relieves residual stress in SiC-to-aluminum brazed joints [55]. Finally, regarding the bulk properties of bismuth-based high-temperature solders, the addition of indium has been shown to refine the microstructure and enhance both tensile strength and ductility [56]. Looking ahead, the integration of these micro-alloying and advanced processing techniques will be critical to developing electronic interconnections capable of withstanding the increasingly extreme thermal and mechanical environments of next-generation power modules.

Advances in additive manufacturing have enabled the development of sophisticated polyamide 12 architectures that transform structural energy absorption and mechanical performance. Researchers first analyzed how basic strut topologies influence performance, discovering that the Octet configuration offers the highest strength and energy absorption among non-auxetic polyamide 12 lattice designs [57]. To improve these truss-based geometries, adding nodal spheres to re-entrant dodecahedral cells effectively reduced stress concentrations and greatly increased compressive stiffness [58]. Beyond geometry, environmental factors like ambient temperature and internal concave angles were found to affect mechanical response, with heat leading to a noticeable decrease in yield strength and energy absorption [59]. Similarly, loading rates are very important because re-entrant chiral auxetic structures are highly sensitive to crushing velocity, often absorbing more energy under quasistatic conditions rather than dynamic ones [60]. Triply periodic minimal surfaces are a class of mathematically defined structures used to create high-performance cellular lattice architectures in engineering. In the intermediate loading range, it was found that entrapped powder within sheet-based triply periodic minimal surfaces can act as secondary reinforcement, preventing the catastrophic failure often seen in strut-based lattices [61]. This idea of secondary materials was intentionally developed through a hybrid material extrusion process that fills structural voids with powder, creating biphasic, architecturally structured materials with customizable functional responses [62]. Further complexity was added by combining carbon fiber reinforcements with hybrid hexagonal and re-entrant layering to create self-sensing honeycombs that maintain high piezoresistive sensitivity even at high temperatures [63]. Lastly, these optimized architectural strategies were applied to practical industrial components, demonstrating that hybrid auxetic structures in dowel pins can reduce mounting forces while increasing resistance to removal [64]. The ongoing integration of complex-geometry optimization and multi-material approaches indicates a future direction where additively manufactured structures can be precisely tailored for adaptive, high-performance engineering applications.

Precise control over surface wettability and interfacial phenomena is essential for advancing applications in thermal management, flexible electronics, and high-performance metallic coatings. A cost-effective spray-coating method using copper nanoparticles was demonstrated to produce microstructured metallic surfaces that promote spontaneous liquid-metal wetting via capillary-driven imbibition [65]. Further research in nanoscale surface engineering revealed that fabricating nanostructures on sintered copper powders enhances hydrophilicity and accelerates drop penetration, thereby improving capillary wicking [66]. A different surface modification strategy described a method for adjusting the morphology of thermally evaporated copper films on liquid substrates, enabling a controlled switch between hydrophilic and hydrophobic states [67]. The reversible spontaneous wetting of Galinstan on copper in alkaline media was studied, with interfacial redox reactions identified as the main cause of spreading and retraction [68]. Multiscale modeling demonstrated that optimal hexagonal patterns of nickel particles can surpass the inherent non-wetting behavior of liquid silver on oxide surfaces [69]. In cooling applications, modified copper surfaces with a wide range of wettability were evaluated, showing that hydrophilicity enhances non-boiling heat transfer, while hydrophobicity favors two-phase regimes in spray cooling [70]. Triangle-array heterogeneous coatings were shown to control the flow of aluminum alloy melts on inclined plates, thereby improving heat transfer and promoting grain refinement [71]. Copper–graphene nanoplatelet composite coatings were also developed, offering superhydrophobicity and enhanced corrosion resistance while promoting efficient dropwise condensation [72]. Finally, superhydrophobic laser-textured surfaces were shown to effectively reduce harmful nanoparticle buildup typically observed during pool boiling with nanofluids [73].

The exploration of strongly correlated electron systems is fundamental to modern materials engineering, as these systems provide the physical basis for developing next-generation technologies such as unconventional superconductors and fault-tolerant quantum computing architectures [74]. Recent studies investigate the complex phase behavior of such systems, specifically focusing on heavy-fermion materials and quantum spin liquids to characterize how localized magnetic moments interact with itinerant electrons to produce emergent states [75]. A primary focus is the Doniach phase diagram, where researchers have developed material-specific, quantitative models for compounds such as CeRu_2_Ge_2_ by analyzing the competition between Kondo screening and Ruderman–Kittel–Kasuya–Yosida interactions. In lower-dimensional systems, studies have explored the Kondo-Heisenberg chain, identifying quantum phase transitions between valence-bond solids and Kondo insulators [76]. Advanced numerical simulations reveal exotic “ultra-local” criticality in 1D Kondo lattice models, characterized by momentum-independent spin fluctuations and infinite dynamical exponents that challenge standard theoretical frameworks [77]. The development of dynamical response theories using random-phase approximations provides a tool for identifying sharp “spinon exciton” features in generic Majorana systems [78]. These works enhance the ability to predict and engineer electronic instabilities in materials under extreme pressure or magnetic fields.

The global push toward sustainable high-performance magnetic materials has catalyzed research into refining the entire life cycle of neodymium-iron-boron (NdFeB) magnets, from fundamental microstructural recovery to advanced additive manufacturing. Understanding the core of this transition begins with low-temperature hydrogenation, where identifying distinct Nd-rich phase transformations and hydride formations is essential for effective pulverization and microstructural recovery of discarded hard disk drive scrap [79]. This recovery process is further enhanced by selectively removing oxidized grain-boundary phases via dry cyclone separation, yielding a near-stoichiometric powder that produces recycled magnets with higher remanence and coercivity [80]. To improve irregular scrap, researchers have created spherical single-crystal precursor powders that, under optimal hydrogen disproportionation pressure, inherit the parent alloy’s strong crystallographic texture, resulting in exceptional energy products [81]. By integrating these refined materials into the digital age, lithography-based composite manufacturing now couples in situ magnetic-field alignment with electron-beam post-curing to overcome UV-light attenuation in high-filler slurries, producing anisotropic bonded magnets with stabilized hysteresis loops and fivefold increases in mechanical strength [82]. For industrial-scale production, powder extrusion molding successfully concentrates magnetic fields within moving strands, achieving high degrees of alignment without post-machining, offering a scalable route to near-net-shape recycled magnets [83]. Alternatively, laser powder bed fusion allows for complex geometries with internal cooling channels, where post-processing heat treatments can improve remanence by up to 5% by reducing porosity and enhancing grain-boundary connectivity [84]. To maximize the density of these printed components, bimodal packing strategies use fine Sm-Fe-N particles to fill the gaps between larger NdFeB particles, increasing the filling fraction to 81 vol%, well beyond the limits of monosized particles [85]. These hybrid designs, when bonded in polyphenylene sulfide via large-area additive manufacturing, not only optimize performance but also ensure superior long-term thermal stability and corrosion resistance in tough environments [86]. Environmental impact is further reduced by using biodegradable polymer matrices combined with recycled NdFeB or rare-earth-free ferrite fillers, showing a manageable move toward a sustainable circular economy [87]. The recycling loop is closed by cryomilling, which pulverizes waste printed magnets into composite powders for direct warm compaction, enabling the efficient reuse of end-of-life materials with minimal performance loss [88]. Combining microstructural accuracy with novel manufacturing and recycling processes will ensure a sustainable, high-performance future for permanent magnet technology.

Recovery of gold and other precious metals from waste electrical and electronic equipment has become increasingly important, and hydrometallurgical methods offer selective, lower-energy, and more sustainable alternatives to pyrometallurgical routes. Multi-step selective acid leaching of a computer’s central processing unit connector pins has been shown to dissolve base metals first, yielding approximately 99% pure gold with only 2–4% loss [89]. A two-step hydrometallurgical extraction using a strong and a weak base anion resin was reported by Sronsri et al., achieving 95.1% gold recovery from e-waste residues [90]. A review of hydrometallurgical methods, i.e., leaching, adsorption, and bio-recovery, focuses on sustainable process options and the challenges of scale-up [91]. Oxidative leaching with hydrogen chloride/sodium hypochlorite at 60 °C has been shown to achieve approximately 91.6% gold extraction, with selective separation from silver and copper [92]. A thorough ionic-liquid-based hydrometallurgical process has been developed to recover gold as nanoparticles with at least 95% purity from leachates of real integrated circuits and central processing units [93]. Triiodide ionic liquids have been developed to allow ultra-fast (about 5 min), room-temperature, selective extraction of over 99.3% gold from e-waste with good recyclability [94]. Emerging trends like metallic organic frameworks, solvent extraction, bio-recovery, and photocatalysis have been reviewed, highlighting advanced selective recovery strategies and their potential sustainability benefits [95]. The study by Ormuž et al. synthesizes recent advances in selective gold recovery from waste electrical and electronic equipment using a speciation-driven approach, critically assessing mechanical pretreatment, physical beneficiation, and hydrometallurgical leaching methods to overcome the drawbacks of conventional pyrometallurgy and non-selective leaching, such as high energy demand, toxic reagents, and complex leach solutions [96].

The same commitment to sustainability and resource efficiency that drives the recovery of precious metals from electronic waste is equally evident in the construction industry, where intensifying pressure to reduce environmental impact has led researchers to explore agricultural byproducts as viable alternatives to conventional concrete aggregates. A review of these efforts shows that various agricultural wastes, including fruit shells and seeds, can effectively substitute traditional aggregates to improve the sustainability and insulation properties of cement composites [97]. One specific application uses date seed aggregates and *Arundo donax* fibers in cement bricks, enhancing the thermal insulation of building envelopes and reducing operational energy use [98]. Studies on olive pomace and mill wastewater further show that these waste streams can partially replace sand in mortar, with a 5% substitution providing a good balance between workability and mechanical strength [99]. Similarly, coconut shell aggregates have been successfully used to create lightweight green concrete that lowers both production costs and carbon dioxide emissions compared to traditional mixes [100]. When these coconut shells are combined with manufactured sand, they can even create self-compacting concrete that meets strict fresh property standards without sacrificing structural integrity [101]. For ecological infrastructure, modified corn cob aggregates processed via a respiratory impregnation method have enabled the production of sustainable pervious concrete with improved frost and sulfate erosion resistance [102]. The boundary of this field now reaches additive manufacturing, where rice husk particles serve as multifunctional aggregates to control the rheology and layer stability of 3D printable cementitious composites [103]. Further decarbonization is achieved by adding wood-residue biochar to fiber cement, serving as a low-carbon design strategy to reduce the overall environmental impact of ordinary Portland cement [104]. To better utilize biomass resources in engineering, researchers are also employing scanning electron microscopy and micro-computed tomography inverse reconstruction to analyze the tripartite structure of sorghum straw, specifically how its nodal microarchitecture resists high stresses and provides hierarchical energy absorption [105]. To ensure the reliability of these materials, advanced imaging techniques like micro-computed tomography and deep learning are now being used to assess the complex microstructural interactions at the interface between bio-aggregates and the cement matrix [106]. The continued integration of such sustainable, bio-based aggregates into diverse construction formats predicts a future in which high-performance infrastructure is linked to circular agricultural practices.

While bio-based aggregates represent a promising pathway to greener construction, the broader pursuit of sustainable energy demands equal attention to the structural integrity of the high-performance turbine components that power modern infrastructure. Understanding the multi-physics degradation of turbine materials is vital for advancing high-efficiency energy systems that must operate reliably under extreme thermo-mechanical loads. This research field is grounded in reviews that categorize failure modes into fatigue, creep, fracture, and vibration-induced damage while highlighting the need for integrated diagnostic strategies [107]. To address these challenges, new high-strength precipitation-hardening steels, such as PH 12-10 Mo, are being developed to provide superior fatigue properties and stress-corrosion cracking resistance for modern large-scale turbine applications [108]. The reliability of turbine steels can be further enhanced by warm pre-stressing, which utilizes specific temperature-load histories to raise fracture resistance through the creation of a residual plastic zone at the crack tip [109]. Detailed numerical models of these effects demonstrate that the integral of the plastic strain magnitude serves as a highly accurate local parameter for predicting fracture loads in steam turbine components. Similarly, extending the warm-up period during the operational start-up phase has been shown to reduce low-cycle fatigue damage in ultra-supercritical steam turbine rotors [110]. Multiphysics investigations of gas turbine blades indicate that thermal stress often dominates pressure forces as the primary driver of crack initiation during start-stop cycles [111]. Material assessments of high-alloyed heat-resistant steels reveal that long-term operation leads to carbide precipitation along grain boundaries, weakening cohesion and reducing fatigue-crack-growth resistance [112]. Coupled mechanical and metallurgical investigations into failed low-pressure blades further identify improper tempering as a root cause of sudden, brittle fractures in high-stress power plant environments [113]. Finally, structural innovations, such as shortening nozzle guide vanes, have been validated through thermal-fluid–structure interaction simulations to effectively reduce localized stresses and improve flow efficiency [114].

The evolution of alumina-based ceramic cutting tools is driven by the need to address their inherent brittleness and low thermal shock resistance through novel material compositions and advanced manufacturing techniques. A review of recent progress shows how alumina-based ceramics continue to dominate the cutting tool market and are being transformed by whiskers, nanoparticles, and advanced sintering techniques to meet modern industrial and sustainability demands [115]. Researchers studying new alumina sintering aids have found that nitinol (Ni-Ti alloy) can serve two purposes: it promotes liquid-phase sintering and acts as a toughening agent that absorbs strain energy through stress-induced phase transformations [116]. In an effort to improve the performance of zirconia-toughened alumina, a comparative study of sintering methods showed that adding copper oxide effectively creates a self-lubricating, soft, thin layer at the tool interface, greatly prolonging tool life [117]. A further investigation into self-lubrication indicates that the incorporation of molybdenum into the zirconia-toughened alumina matrix results in the formation of lubricating molybdenum oxide tribo-films (MoO_2_ and MoO_3_), which effectively resist abrasion during high-speed dry turning [118]. Practical testing in demanding environments shows that both whisker-reinforced alumina and specialized tungsten carbide-reinforced alumina tools outperform traditional coated carbides when turning tough aerospace superalloys like Inconel 718 [119]. Regarding carbide reinforcements in zirconia-toughened alumina, it was found that although tungsten carbide provides superior bulk hardness, zirconium carbide offers better wear resistance against steel as a result of the formation of a stable, iron-enriched protective tribo-film, which is facilitated by its inherent disordered graphitic carbon [120]. Moving to manufacturing innovation, vat photopolymerization is now used to produce alumina inserts with previously impossible chip-breaking grooves, thus lowering cutting temperatures and forces when machining cast iron [121]. Further pushing the limits of 3D printing, a pioneering design incorporates an internal cooling channel filled with liquid gallium to greatly reduce tool wear by improving heat dissipation without relying on external mechanical pumps [122]. Beyond simple mixtures, the development of a new three-layer structured bionic alumina-based cutting insert applies the concept of anisotropy found in seashells to achieve high fracture toughness by combining interlayer cracks with complex crack-propagation mechanisms, such as bridging and pull-out [123]. This ongoing cooperation between bionic materials science and precision additive manufacturing indicates a future in which cutting tools are transformed from static blocks into intelligent, internally cooled, and biologically inspired machining systems.

The fourteen contributions gathered in the Special Issue “Advanced Materials and Processing Technologies” and the Editorial’s discussion of recent developments, knowledge gaps, and future research directions show that progress in materials science is closely linked to innovation in processing materials and a deeper understanding of structure–properties relationships. Throughout the topics covered, a common theme emerges: targeted control of processing conditions, whether thermal, mechanical, chemical, or digital, is the primary way to improve material performance. Looking ahead, the growing integration of digital design tools, data-driven optimization, and sustainable manufacturing strategies is likely to accelerate this progress, ensuring that the next generation of materials and processing technologies meets both the performance needs and the environmental responsibilities of modern engineering.
